# All-Dielectric Huygens’ Metasurface for Wavefront Manipulation in the Visible Region

**DOI:** 10.3390/ma14205967

**Published:** 2021-10-11

**Authors:** Tiesheng Wu, Zhihui Liu, Weiping Cao, Huixian Zhang, Dan Yang, Zuning Yang

**Affiliations:** 1 Guangxi Key Laboratory of Wireless Broadband Communication and Signal Processing, School of Information and Communication, Guilin University of Electronic Technology, Guilin 541004, China; tieshengw@163.com (T.W.); weipingc@guet.edu.cn (W.C.); hxzhang_guet@163.com (H.Z.); dyang_guet@163.com (D.Y.); zuning_yang@163.com (Z.Y.); 2 Key Laboratory of Optoelectronic Devices and Systems of Ministry of Education and Guangdong Province, Shenzhen University, Shenzhen 518060, China; 3 Guangdong and Hong Kong Joint Research Centre for Optical Fiber Sensors, College of Optoelectronic Engineering, Shenzhen University, Shenzhen 518060, China

**Keywords:** Huygens’ metasurface, high transmission, visible region

## Abstract

All-dielectric Huygens’ metasurfaces have been widely used in wavefront manipulation through multipole interactions. Huygens’ metasurfaces utilize the superposition between an electric dipole and a magnetic dipole resonance to realize transmission enhancement and an accumulated 2π phase change. Benefiting from this unique property, we design and numerically investigate an all-dielectric Huygens’ metasurface exhibiting high-efficiency anomalous refraction. To suppress the substrate effect, the metasurface structure is submerged in a dielectric plate. We strategically placed two elements in four short periods to form a unit cell and adjusted the spacing between the two elements to effectively inhibit the interaction between elements. At the operating wavelength of 692 nm, the obtained anomalous transmission efficiency is over 90.7% with a diffraction angle of 30.84°. The performance of the proposed structure is far superior to most of the existing phase-gradient metasurface structures in the visible region, which paves the way for designing efficient beam deflection devices.

## 1. Introduction

Over the recent years, metasurfaces imparting abrupt interfacial phase changes at subwavelength scales have garnered considerable interest from the optics community. Metasurfaces offer a much better platform for beam shaping and electromagnetic wavefront engineering at the nanoscale level. Thus, the functionalities and applications of metasurfaces have been extensively investigated, such as anomalous refractors, metalens, holograms, and vortex beam exciters [1,2,3,4,5,6,7,8,9]. Metasurfaces can perform a wide range of functions through a specific arrangement of phase. Specifically, the ability of metasurfaces to realize optical manipulation at the nanoscale level has facilitated the development of advanced optical systems.

According to the mechanisms of phase control, metasurface can be roughly divided into three categories. The first category is the transmission phase metasurface. Each element of the metasurface can be regarded as a Fabry-Perot cavity with low-quality factor, and the light bounces back and forth between the ends of these structures. Therefore, this type of metasurface is also known as a Fabry-Perot type metasurface [10,11,12,13,14]. However, Fabry-Perot cavities tend to be very long to achieve the conditions for resonance and phase changes. Thus, the high aspect ratios often limit the accurate fabrication of this kind of metasurface. The second category is the geometric (Pancharatnam-Berry) phase metasurface [15,16,17] in which the rotation angle and position of the elements are adjusted to obtain a phase shift. However, the application of such metasurfaces is limited by the special excitation condition, which requires circularly polarized light. The last category is the Huygens’ metasurfaces mentioned in this paper [18,19,20,21,22,23]. Huygens’ metasurfaces achieve nearly 2π phase change through the superposition of electric dipole (ED) and magnetic dipole (MD) resonances. Each Mie resonance can result in an approximate π phase change, and the phase change accumulates when the electric and magnetic resonances of the same order overlap, leading to an approximate 2π phase change. The overlapping of the ED and MD resonances causes directional scattering and enhances the transmission [24]. Compared with other kinds of metasurfaces, the excitation conditions of the Huygens’ metasurfaces are broad. Moreover, the elements of Huygens’ metasurfaces do not require a high aspect ratio, which is more convenient for experimental fabrication. The enhanced transmission due to spectral overlapping makes the design of high-efficiency optical devices more flexible.

However, the design of Huygens’ metasurface often suffers from some problems. For example, the phase profiles and transmissive responses of such metasurfaces are sensitive to the interaction of neighboring elements. The interference of the dipole sources with the metasurface elements destroys the predesigned phase profile. Furthermore, the introduction of a substrate affects the distribution of induced charges and circular currents [25], which changes the far-field radiation patterns of the ED and MD resonances. Therefore, the anomalous transmission efficiency may decline sharply. Zhao et al. designed an all-dielectric metasurface without the substrate, and they assumed that the metasurface is placed in the free space [26]. However, without the support of a substrate, this metasurface may not be practically feasible. Liu et al. designed a phase gradient Huygens’ metasurface with the anomalous transmission efficiency approaching 80% when no substrate was introduced [27]. When the substrate was introduced, the anomalous transmission efficiency rapidly decreased to just 35%. To effectively suppress the substrate effect, they added a low refractive index buffer layer between the metasurface and the substrate. Finally, the anomalous transmission efficiency reached 76%, which verified the effectiveness of this method. However, a drawback of their work is that they used six elements to maintain phase continuity, making the anomalous transmission angle very small and significantly limiting the practical applications. Liu et al. also proposed a three-element Huygens’ metasurface operating in the near-infrared region and achieved an anomalous transmission efficiency of 83.5% by covering the metasurface elements with a coating whose refractive index was close to that of the substrate [28]. However, the absorption of silicon materials is negligible and can be ignored in the near-infrared region, but their absorption loss in the visible region cannot be ignored. In addition, although the extinction coefficient of titanium dioxide, another commonly used dielectric material, is very low, its refractive index is also very low, which often requires a high aspect ratio. To solve these problems, we first propose the use of aluminum antimonide to design metasurfaces. When the wavelength is greater than 560 nm, the refractive index of the aluminum antimonide is very close to that of silicon, but its extinction coefficient is much less than that of silicon, and the absorption loss can be effectively reduced. To effectively suppress the anomalous transmittance reduction caused by the substrate effect, the metasurface structure is submerged in a sufficiently thick polymer-based dielectric plate.

In this work, we have achieved a high transmission efficiency of Huygens’ metasurface with anomalous refraction by the spectral overlap of ED and MD resonances. Since the silicon material has some absorption loss in the visible region, we use aluminum antimonide instead. When the wavelength is greater than 560 nm, the refractive index of the aluminum antimonide is very close to that of silicon, but its extinction coefficient is much less than that of silicon. To suppress the substrate effect, the metasurface structure is submerged in a low refractive index dielectric plate to create a photonic-crystal slab. We strategically placed two elements in four short periods to form a unit cell and adjusted the spacing between the two elements to effectively inhibit the interaction between elements. The results show that the anomalous transmission efficiency is 90.7% with a diffraction angle of 30.84° at the operating wavelength of 692 nm. Further, nearly 95% of the transmitted light can be directed along the desired refractive angle. The performance of this metasurface is much better than that of the existing phase-gradient metasurface structures in the visible region. Thus, the proposed design strategy is potentially beneficial for the design of highly efficient beam deflection devices.

## 2. Design Strategy

### 2.1. Electromagnetic Multipole Decomposition Method

It is well known that in addition to the ED modes, high-order electric multipole and magnetic multipole modes can be excited in a scatterer by light. These modes can significantly affect the optical properties of structural materials and even produce strange optical phenomena, such as negative refraction [29]. The decomposition of scattered light from the nanoparticles into a series of multipole modes is of great significance for examining the light excitation and regulation at the nanoscale level. Here, we mainly utilize a Cartesian coordinate decomposition method [30,31,32,33,34]. This method does not directly decompose the scattered field in Cartesian coordinates. Instead, the multipole moment is calculated based on the electric field inside the particles. Then, the scattering intensity of the multipole is calculated to obtain the scattering cross-section. The scattering cross-section is calculated based on the commercial simulation software COMSOL Multiphysics 5.5 [35]. The electromagnetic wave (EMW) frequency domain model under the radio frequency module is used to calculate the scattering cross-section. The upper and lower interfaces are set as scattering boundary conditions. The input port is set below and the output port is set above the structure. The boundary conditions for a perfect electric conductor and a perfect magnetic conductor are set in the x- and y-directions, respectively. The mesh is a physics-controlled mesh, and the mesh size is set to extremely fine. The origin of the Cartesian coordinate system coincides with the center of mass of the nanoparticle. The multipolar moments of the particle are defined as [31,32]

(1) $$p=\varepsilon_{0}\left(\varepsilon_{s}-\varepsilon_{p}\right)\int E\left(r\right)d^{3}r$$

(2) $$m=\frac{i\omega \varepsilon_{0}\left(\varepsilon_{s}-\varepsilon_{p}\right)}{2v_{s}}\int \left(r\times E\left(r\right)\right)d^{3}r$$

where *ε_0_*, *ε_s_*, *ε_p_*, *v_s_*, and ***E***(***r***) are the vacuum dielectric constant, relative dielectric permittivity of the surrounding medium, relative dielectric permittivity of the nanoparticle, velocity of light in the surrounding medium, and the total electric field at position ***r*** inside the particle, respectively. Multipole decomposition integration is performed over the scatterer volume. Then, we can obtain the scattering power of the ED and MD as follows [33,34]:

(3) $$P_{p}=\frac{k_{0}^{4}}{12\pi \varepsilon_{0}^{2}v_{s}\mu_{0}}{\left|p\right|}^{2}$$

(4) $$P_{m}=\frac{k_{0}^{4}\varepsilon_{s}}{12\pi \varepsilon_{0}v_{s}}{\left|m\right|}^{2}$$

where *k*_0_ and *μ*_0_ are the wavenumber in vacuum and the vacuum permeability, respectively. Further, the scattering cross-sections are defined in terms of the scattering power normalized to the energy of the incident light $\;I_{inc}={\left(\varepsilon_{0}\varepsilon_{d}/\mu_{0}\right)}^{1/2}{\left|E_{inc}\right|}^{2}/2$,

(5) $$\sigma_{p}=\frac{k_{0}^{4}}{6\pi \varepsilon_{0}^{2}{\left|E_{inc}\right|}^{2}}{\left|p\right|}^{2}$$

(6) $$\sigma_{m}=\frac{k_{0}^{4}\varepsilon_{d}\mu_{0}}{6\pi \varepsilon_{0}{\left|E_{inc}\right|}^{2}}{\left|m\right|}^{2}$$

### 2.2. Metasurface Design

**Figure 1:**
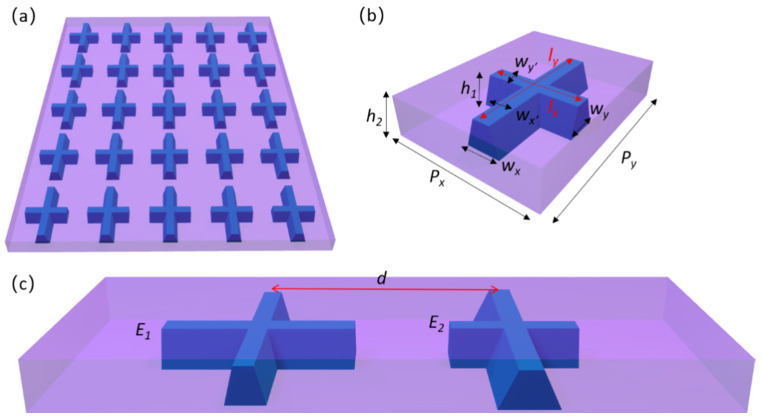
(**a**) Schematic of the periodic cross-shaped structure. (**b**) Schematic of a single cell. The parameters of each variable of the metasurface are labeled. (**c**) Schematic of the proposed phase-gradient metasurface. The two elements are marked as *E*_1_ and *E*_2_. The detailed parameters are shown in Table 1.

To achieve the overlap of ED and MD resonances, we design and numerically investigate cross-shaped structures with a trapezoidal cross-section. A schematic of the proposed cross-shaped structure is presented in Figure 1a. Aluminum antimonide is used as the material for the metasurface, and the optical constants are taken from Ref. [36]. In addition, the proposed structure is embedded into a dielectric plate made of tetrafluoroethylene hexafluoropropylene vinylidene (THV) with a refractive index of 1.35. Here, the commercially available software Lumerical FDTD is used to numerically model the optical properties [37]. The periodic boundary conditions are set along the x- and y-directions, while the perfectly matched layer is set along the z-direction. Further, a linearly x-polarized light is normally incident on the bottom. The mesh size is 5 nm along the x-, y-, and z-directions near the metasurface. The mesh size along the z-direction in the air is set to 20 nm. By placing power monitors below the light source and above the structures, we can obtain the normalized reflectivity and transmittance, respectively. As shown in Figure 1b, the period of a single cell along the x- and y-directions is set to 340 and 450 nm, respectively. The length *l_x_*, *l_y_*, and the width *w_x_*, *w_y_* are 200, 320, 70, and 60 nm, respectively, and the thicknesses *h_1_* and *h*_2_ are set to 200 and 250 nm. The ratio of *w_x’_* to *w_x_* is defined as *k*_1_. Similarly, the ratio of *w_y’_* to *w_y_* is denoted by *k*_2_. Here, the value of *k_1_* and *k_2_* are both 0.5. Unless otherwise specified, subsequent analyses are mainly based on these geometric parameters.

**Figure 2:**
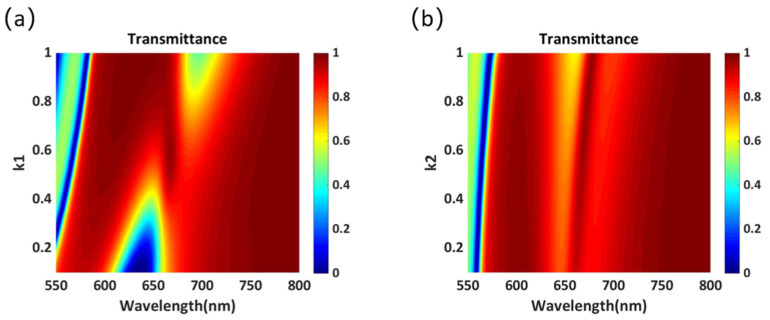
Transmittance of the proposed cross-shaped structure as a function of (**a**) *k*_1_ and (**b**) *k*_2_.

For the design of phase-gradient metasurfaces, there are two important conditions: 2π phase control and high transmittance. Therefore, the effects of each parameter on transmittance and phase must be examined. The transmission spectrum of the cross-shaped structure in Figure 1a as a function of *k*_1_ and *k*_2_ are shown in Figure 2a,b, respectively. It is evident that when *k*_1_ is close to 0.5, a higher transmittance can be obtained in the wavelength range of 600–700 nm. Further, when *k*_1_ is greater than 0.8, the transmittance decreases to less than 60% in the wavelength range of 650–700 nm. However, the transmittance is insensitive to the variation in *k*_2_. Especially, when the wavelength is greater than 660 nm, the transmittance can exceed 80% with the variation in *k_2_*.

**Figure 3:**
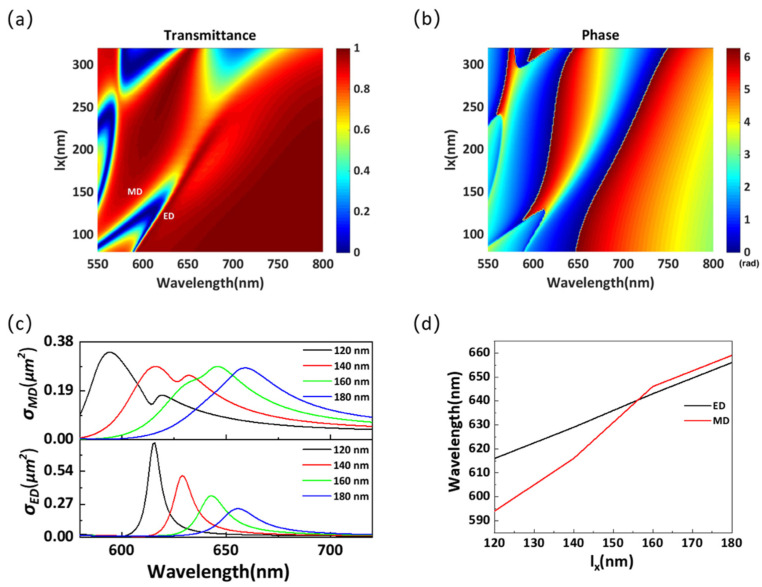
(**a**) Transmittance and (**b**) phase for different structural parameters *l_x_* in the wavelength range of 550–800 nm. (**c**) Scattering cross-section for different structural parameters *l_x_* in the wavelength range of 550–800 nm. (**d**) Resonant wavelengths of Mie resonances for different structural parameters *l_x_*.

Figure 3a,b shows the variation in the transmittance and phase of the proposed structure as a function of the length *l_x_* in the wavelength range of 550–800 nm. It can be seen in Figure 3a that there are two distinct transmission valleys in the wavelength range of 550–650 nm. The wavelengths of these valleys exhibit a redshift with the increase in *l_x_*. However, they move on different slopes, overlapping at a wavelength of nearly 640 nm, causing enhanced transmission. As shown in Figure 3b, after the overlap of two transmission valleys, the phase shift of the transmitting light becomes 2π. To demonstrate the complete 2π phase control mechanism, the contributions of two dipole modes at different *l_x_* to the scattering cross-section are calculated using the electromagnetic multipole decomposition method, and the results are shown in Figure 3c. It is clear that the MD mode exhibits a faster redshift than the ED mode, and it gradually exceeds the ED mode with the increase in *l_x_*. Figure 3d shows the variations in the resonant wavelengths of two Mie resonances as a function of *l_x_*. According to Figure 3d, the two modes overlap at a wavelength of 640 nm. The results are consistent with those in Figure 3a,b, proving that the 2π phase control mechanism is based on the overlap of the ED and MD resonances. Furthermore, when the wavelength is close to 550 nm, two more obvious transmission valleys appear in Figure 3a due to the excitation of higher-order Mie resonances.

**Figure 4:**
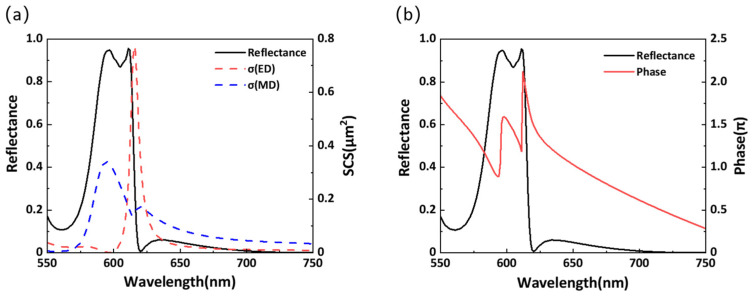
(**a**) Reflectance and multipolar decomposition of scattering cross-sections in the wavelength range of 550–750 nm when *l_x_* is 120 nm. (**b**). Reflectance and phase in the wavelength range of 550–750 nm.

**Figure 5:**
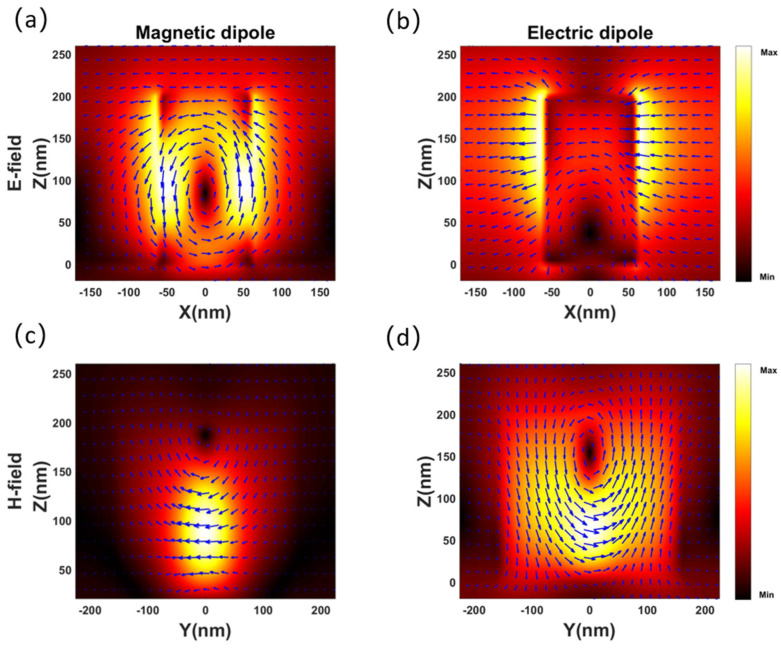
Simulated electric field magnitude distributions ${\left|E\right|}^{2}$ and vector distributions of the (**a**) MD and (**b**) ED resonances in the x-z plane. Simulated magnetic field magnitude distributions ${\left|H\right|}^{2}$ and vector distributions of the (**c**) MD and (**d**) ED resonances in the y-z plane.

Figure 4a shows the calculated reflectance in the wavelength range of 550–750 nm for *l_x_* = 120 nm. For comparison, the multipolar decomposition of scattering cross-sections in terms of ED and MD are also shown as red and blue dashed lines, respectively. There are two distinguishable reflection peaks at the wavelengths of 579 and 612 nm, which are highly consistent with the resonance wavelengths of ED and MD. This proves that the two reflection peaks are excited by Mie resonances and unambiguously reveals the independent tuning of the dipole resonance. To further clarify this phenomenon, the phase change of the transmitted light is shown in Figure 4b. It is found that the phase changes by approximately π when the ED or MD resonance is excited. As the value of *l_x_* increases, the phase change accumulates when the ED and MD resonances overlap, resulting in an approximate 2π phase change. To further prove that Mie resonances cause the two reflection peaks, the electric field distributions (Figure 5a,b) and magnetic field distributions (Figure 5c,d) at the two reflection peaks are obtained. Based on the vector distribution, circular and linear displacement currents can be identified more intuitively and clearly, indicating the MD and ED resonances, respectively.

**Figure 6:**
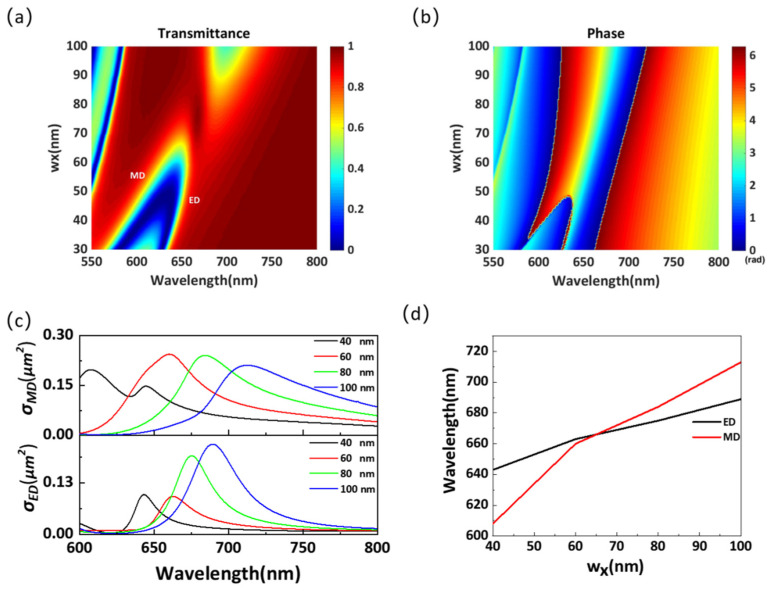
(**a**) Transmittance and (**b**) phase for different structural parameters *w_x_* in the wavelength range of 550–800 nm. (**c**) Scattering cross-section for different structural parameters *w_x_* in the wavelength range of 550–800 nm. (**d**) Resonant wavelengths of Mie resonances for different structural parameters *w_x_*.

Figure 6a,b shows the variation in the transmittance and phase of the proposed structure as a function of the width *w_x_* in the wavelength range of 550–800 nm. It can be seen that the two distinct transmission valleys exhibit a redshift with the increase in *w_x_* and overlap at a wavelength of approximately 660 nm when *w_x_* is nearly 65 nm. Similar to Figure 3a,b, the overlap of two transmission valleys results in a 2π phase shift. Figure 6c,d shows the results of the electromagnetic multipole decomposition in the wavelength range of 600–800 nm. It is clear that with the increase in *w_x_*, the ED and MD eventually overlap at a wavelength of approximately 660 nm. Further, the overlapping wavelength of the ED and MD is different from that in Figure 3d, proving that the overlapping wavelength can be dynamically controlled by *l_x_* and *w_x_*.

**Figure 7:**
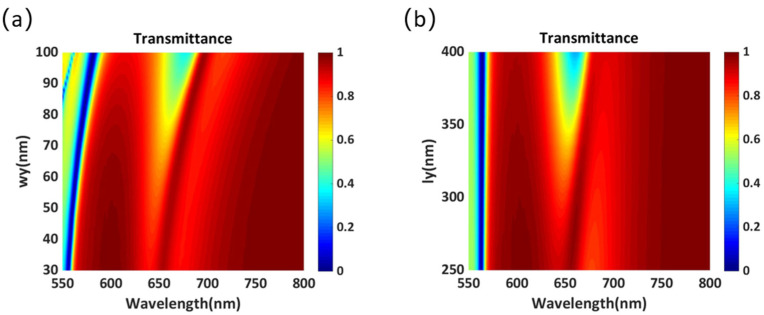
Transmittance of the proposed cross-shaped structure as a function of (**a**) *w_y_* and (**b**) *l_y_*.

Figure 7a,b shows the influence of *w_y_* and *l_y_* on the transmittance. There is a transmission valley near the wavelength of 550 nm, which may be excited by high-order Mie resonance. However, this resonance mode is insensitive to the change in *w_y_* and *l_y_*. Furthermore, in the wavelength range of 600–700 nm, no obvious dipole resonances are excited with the variation in *w_y_* and *l_y_*, which also indicates that the phase of the transmitted light does not change significantly with the change in these two parameters. However, by adjusting these two parameters, a higher transmittance can be obtained.

According to the above analysis, it can be inferred that *l_x_* and *w_x_* are the two parameters that greatly influence the overlap of the ED and MD resonances. Therefore, *l_x_* and *w_x_* are considered as the main optimization parameters to design a two-element phase-gradient Huygens’ metasurface. Compared with some earlier reports [11,12,13,18,26,27,28], the two-element metasurface has fewer optimization parameters, which simplifies the design process of the metasurface. To effectively inhibit the interaction between elements and obtain a large anomalous refraction angle, two elements are strategically placed in four short periods to form a unit cell, as shown in Figure 1c. The periodicity of the metasurface along the x- and y-directions is set as 1350 and 450 nm, respectively. The two elements are named as *E*_1_ and *E*_2_. The space between the two elements is denoted by *d*. To achieve the highest transmission efficiency, the geometrical parameters are optimized, and the detailed parameters are shown in Table 1.

## 3. Results and Discussion

**Figure 8:**
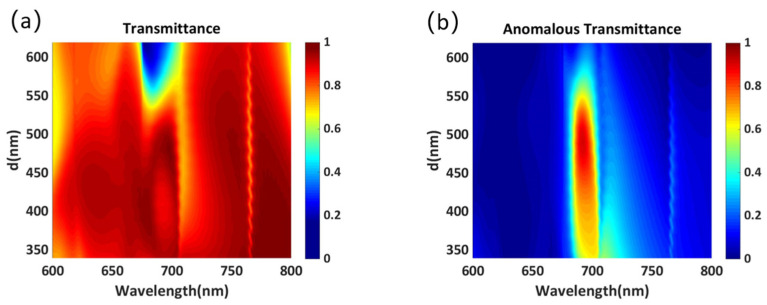
(**a**) Transmittance and (**b**) anomalous transmittance as a function of the element spacing *d*.

**Figure 9:**
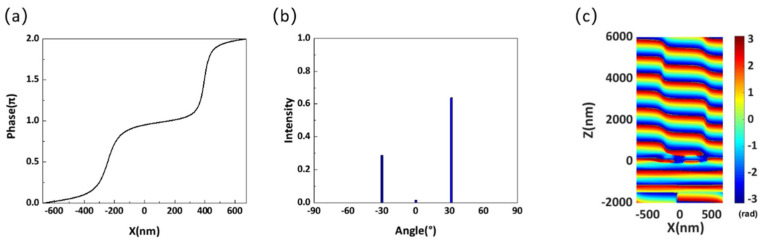
(**a**) Simulated phase distribution along the x-direction at the wavelength of 702 nm with element spacing *d* = 340 nm. (**b**) Far-field transmission intensity for different diffraction orders at the wavelength of 702 nm. (**c**) Phase distribution of the metasurface with an element spacing of 340 nm at the wavelength of 702 nm.

The influence of element spacing on the transmittance and anomalous transmittance is simulated to reduce the effect of the interaction between neighboring Huygens’ elements. As shown in Figure 8a,b, when the spacing *d* between two elements is too large, the transmittance and anomalous transmittance at the central wavelength decrease sharply. For example, when the spacing is 600 nm, the anomalous transmittance drops to nearly 27%. When the spacing *d* is small, higher transmittance can be obtained in the entire wavelength range, but the anomalous transmission efficiency at the center wavelength is reduced by the interaction between Huygens’ elements. Figure 9a shows the simulated phase distribution along the x-direction at the wavelength of 702 nm with an element spacing of 340 nm. Although a complete 2π phase shift can be realized in the unit cell, the phase change is not smooth enough due to the interaction between Huygens’ elements. Figure 9b shows the far-field transmission intensity for different diffraction orders. Although the total transmittance can reach 94.5%, only 63.9% of the energy can be deflected to the +1 diffraction order due to the interaction between Huygens’ elements. Further, nearly 30% of the energy is mainly concentrated in the −1 diffraction order, which limits the performance of the metasurface. Figure 9c illustrates the phase distribution of the transmitted light in the x-z plane, and it is obvious that the wavefront is not smooth. As shown in Figure 8b, the maximum anomalous transmittance can be obtained when the spacing *d* is close to 490 nm. Therefore, to reduce the interactions between Huygens’ elements and achieve high performance, the spacing was adjusted to 487 nm.

**Figure 10:**
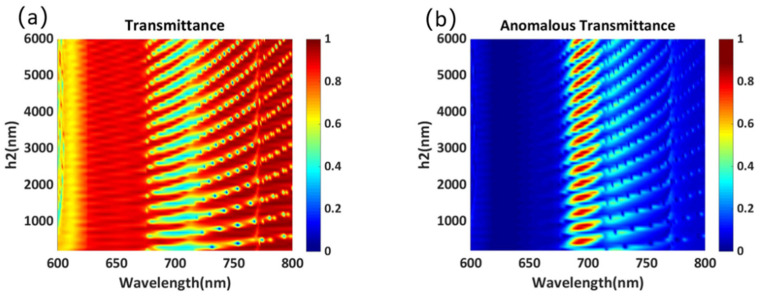
(**a**) Transmittance and (**b**) anomalous transmittance as a function of the thickness *h_2_*.

The thickness of the dielectric plate is crucial to the performance of the metasurface and the actual manufacturing process. As shown in Figure 10a,b, we simulated the variation in the transmittance and anomalous transmittance with the thickness of the polymer-based dielectric plate. It can be seen that the peak values of transmittance and anomalous transmittance change periodically with the increase in the thickness of the dielectric plate, and the variation period is close to the working wavelength. In addition, with the increase in the thickness of the dielectric plate, the peak value of anomalous transmittance can reach more than 90%. Here, to facilitate calculation, the dielectric plate is set to 250 nm. The dielectric plate can be made as thick as possible in real fabrication, and we believe that high anomalous transmittance can still be obtained.

**Figure 11:**
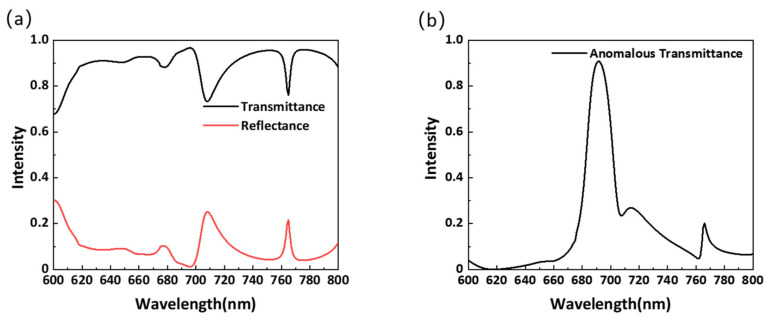
(**a**) Simulated intensities of the transmitted and reflected light. (**b**) Simulated intensity of the anomalous transmittance.

Figure 11a shows the calculated transmittance and reflectivity curves of the optimized structure in the wavelength range of 600–800 nm. The transmittance is high and can be maintained at more than 70% over the entire wavelength range. As the spacing is adjusted, the wavelength of the highest transmittance is shifted from 702 nm to 692 nm. When the wavelength is 692 nm, the total transmittance can reach 95.8%, and the reflectivity is 1.9%. It can be seen that the decrease in transmittance is mainly caused by the increase in reflectance, and the sum of the two is nearly 1 at most wavelengths, which proves that the absorption of the structure is negligible. The calculation results reveal that the absorption rate in the entire band is less than 2.5%, which proves that aluminum antimonide is an appropriate material for the design of metasurfaces as it can effectively reduce the absorption loss. In addition, the transmittance decreases significantly when the wavelength is less than 620 nm. This is due to the reflectivity enhancement caused by the excitation of higher-order Mie resonances. Figure 11b shows the anomalous transmittance in the wavelength range of 600–800 nm. The anomalous transmittance can reach as high as 90.7% at the central wavelength of 692 nm.

**Figure 12:**
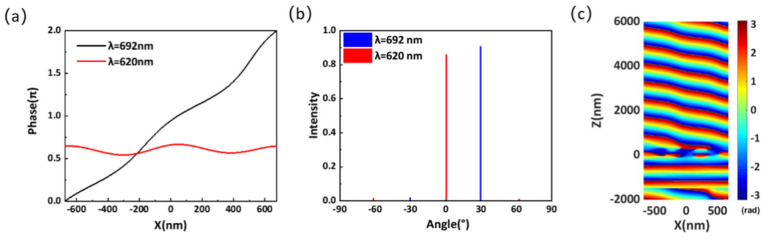
(**a**) Simulated phase distribution of the optimized structure along the x-direction at the wavelengths of 620 and 692 nm. (**b**) Far-field transmission intensity for different diffraction orders at the wavelengths of 620 and 692 nm. (**c**) Phase distribution of the optimized metasurface at the wavelength of 692 nm.

Figure 12a shows the simulated phase distributions along the x-direction at the wavelengths of 620 and 692 nm. It is clear that the phase shift is very smooth after adjusting the spacing at the wavelength of 692 nm, and the phase does not cover the complete 2π range at the wavelength of 620 nm. Figure 12b indicates that the energy of transmitted light is mainly concentrated in the +1 diffraction order with a diffraction angle of 30.84°, and the anomalous transmittance can reach as high as 90.7% at the wavelength of 692 nm. Because the phase does not cover the complete 2π range, the metasurface is unable to achieve anomalous transmission, and the majority of transmitted light is concentrated at 0°. Furthermore, the phase distribution of the transmitted light in the x-z plane at the wavelength of 692 nm is shown in Figure 12c. It can be seen that after adjusting the element spacing, the wavefront of the transmitted light becomes smooth, which validates the efficacy of our design strategy.

## 4. Conclusions

We designed and numerically investigated an all-dielectric Huygens’ metasurface capable of high-efficiency anomalous transmission. Using the electromagnetic multipole decomposition method, it was demonstrated that the excitation of the ED and MD resonances was closely related to the geometric parameters *l_x_* and *w_x_*. By adjusting the parameters to realize the overlapping of the ED and MD resonances, high transmission and a complete 2π phase shift were achieved. To suppress the substrate effect, the metasurface elements were submerged in a polymer-based dielectric plate. Meanwhile, to reduce the interaction between the Huygens’ elements and obtain a larger diffraction angle, the two elements were strategically placed in four short periods to form a unit cell. Furthermore, high anomalous transmittance could be obtained by adjusting the element spacing *d*. The maximum anomalous transmittance was 90.7% with a diffraction angle of 30.84° at the operation wavelength of 692 nm. Further, nearly 95% of the transmitted light could be directed along the desired refractive angle. The performance of the proposed structure is superior to most of the previously reported structures. Accordingly, we believe that the proposed design strategies may play a vital role in the design of highly efficient beam deflection devices.

## Figures and Tables

**Table 1:** Crucial Parameters of the Huygens’ Metasurface.

Element	*l_x_* (nm)	*w_x_* (nm)	*l_y_* (nm)	*w_y_* (nm)	*w_x’_* (nm)	*w_y’_* (nm)
*E*_1_	197	67	385	94	36	46
*E*_2_	301	79	344	60	35	30

## Data Availability

Data sharing is not applicable to this article.
